# Involvement of AMPA Receptor GluR2 and GluR3 Trafficking in Trigeminal Spinal Subnucleus Caudalis and C1/C2 Neurons in Acute-Facial Inflammatory Pain

**DOI:** 10.1371/journal.pone.0044055

**Published:** 2012-08-24

**Authors:** Makiko Miyamoto, Yoshiyuki Tsuboi, Kuniya Honda, Masayuki Kobayashi, Kogo Takamiya, Richard L. Huganir, Masahiro Kondo, Masamichi Shinoda, Barry J. Sessle, Ayano Katagiri, Daiju Kita, Ikuko Suzuki, Yoshiyuki Oi, Koichi Iwata

**Affiliations:** 1 Department of Anesthesiology, Nihon University School of Dentistry, Kandasurugadai, Chiyoda-ku, Tokyo, Japan; 2 Department of Physiology, Nihon University School of Dentistry, Kandasurugadai, Chiyoda-ku, Tokyo, Japan; 3 Division of Functional Morphology, Dental Research Center, Nihon University School of Dentistry, Kandasurugadai, Chiyoda-ku, Tokyo, Japan; 4 Department of Oral and Maxillofacial Surgery, Nihon University School of Dentistry, Kandasurugadai, Chiyoda-ku, Tokyo, Japan; 5 Department of Pharmacology, Nihon University School of Dentistry, Kandasurugadai, Chiyoda-ku, Tokyo, Japan; 6 Department of Medical Sciences, Section of Integrative Physiology Faculty of medicine, Graduate School of Medicine University of Miyazaki, Kihara, Kiyotake-cho, Miyazaki-shi, Miyazaki, Japan; 7 Department of Neuroscience, Howard Hughes Medical Institute, Johns Hopkins University School of Medicine, Baltimore, Maryland, United States of America; 8 Department of Physiology, University of Toronto, Toronto, Ontario, Canada; Kaohsiung Chang Gung Memorial Hospital, Taiwan

## Abstract

To evaluate the involvement of trafficking of alpha-amino-3-hydroxy-5-methyl-4-isoxazolepropionic acid receptor (AMPAR) GluR2 and GluR3 subunits in an acute inflammatory orofacial pain, we analyzed nocifensive behavior, phosphorylated extracellular signal-regulated kinase (pERK) and Fos expression in Vi/Vc, Vc and C1/C2 in GluR2 delta7 knock-in (KI), GluR3 delta7 KI mice and wild-type mice. We also studied Vc neuronal activity to address the hypothesis that trafficking of GluR2 and GluR3 subunits plays an important role in Vi/Vc, Vc and C1/C2 neuronal activity associated with orofacial inflammation in these mice. Late nocifensive behavior was significantly depressed in GluR2 delta7 KI and GluR3 delta7 KI mice. In addition, the number of pERK-immunoreactive (IR) cells was significantly decreased bilaterally in the Vi/Vc, Vc and C1/C2 in GluR2 delta7 KI and GluR3 delta7 KI mice compared to wild-type mice at 40 min after formalin injection, and was also significantly smaller in GluR3 delta7 KI compared to GluR2 delta7 KI mice. The number of Fos protein-IR cells in the ipsilateral Vi/Vc, Vc and C1/C2 was also significantly smaller in GluR2 delta7 KI and GluR3 delta7 KI mice compared to wild-type mice 40 min after formalin injection. Nociceptive neurons functionally identified as wide dynamic range neurons in the Vc, where pERK- and Fos protein-IR cell expression was prominent, showed significantly lower spontaneous activity in GluR2 delta7 KI and GluR3 delta7 KI mice than wild-type mice following formalin injection. These findings suggest that GluR2 and GluR3 trafficking is involved in the enhancement of Vi/Vc, Vc and C1/C2 nociceptive neuronal excitabilities at 16–60 min following formalin injection, resulting in orofacial inflammatory pain.

## Introduction

The alpha-amino-3-hydroxy-5-methyl-4-isoxazolepropionic acid receptor (AMPAR) consists of a hetero-tetrametric combination of 4 receptor subunits, GluR1-4 [1], and is known to exert its actions as complexes of subunits, mainly GluR1/2 and GluR2/3 [2]. GluRs1-4 are highly expressed in the spinal dorsal horn (DH), and are thought to be involved in somatosensory processing [3]. In an animal peripheral inflammatory pain model, the N-ethylmaleimide-sensitive fusion protein (NSF) was reported to be involved in central sensitization of spinal cord neurons through a GluR2 subunit composition switch following peripheral inflammation [4]. It has also been reported that interactions between the GluR2 C-terminus and its binding proteins regulate receptor internalization in spinal DH neurons in an inflammatory pain model [5]. Phosphorylation of the GluR2 C-terminus by protein kinase C (PKC) regulates these protein bindings. GluR2 is also known to be internalized via NMDA receptor-triggered PKC activation in DH neurons following persistent inflammation of the hind paw [5], [6]. Sequential studies using AMPAR subunit knockout (KO) mice have also shown that GluR2 enhances nociceptive plasticity, resulting in the enhancement of inflammatory hyperalgesia, whereas GluR1 KO mice show subtle abnormalities in the inflammatory pain [7], [8]. This indicates that the GluR2 subunit may be involved in persistent pain following peripheral inflammation.

GluR3 as well as GluR2 are also known to be expressed throughout the grey matter of the spinal cord, although GluR2 is relatively restricted in laminae I-II of the spinal DH, whereas GluR3 expression is distributed more abundantly in laminae I and III [9]. Both GluR2 and GluR3 have an identical C-terminal amino acid sequence (SVKI, class II PDZ domain ligand sequence). The GluR2 subunit has been known to have specific interactions with intracellular molecules determining AMPAR functions such as trafficking, synaptic plasticity and dendritic spine formation [10], [11]. The glutamate receptor interacting protein (GRIP) 1/2 and protein interacting C kinase (PICK) 1 are also reported as interacting proteins with these sequences [12]. These findings indicate that GluR2 and GluR3 are important subunits involved in somatosensory processing in the spinal DH. However, the functional role of GluR3 is not known and the involvement of GluR2/3 associated with peripheral inflammation is still unclear.

It is well known that orofacial noxious stimulation activates second-order nociceptive neurons (both wide dynamic range [WDR] and nociceptive-specific neurons) in the trigeminal spinal subnucleus interpolaris (Vi) and subnucleus caudalis (Vc) transition zone (Vi/Vc), Vc and upper cervical spinal cord (C1/C2), and nociceptive neurons receiving noxious inputs from the orofacial region are somatotopically organized in these regions [13]–[15]. Following orofacial inflammation, the neuronal excitability of Vi/Vc, Vc and C1/C2 nociceptive neurons is significantly enhanced and the receptive fields of these nociceptive neurons are significantly increased [16]–[18]. Furthermore, the extracellular signal-regulated kinase (ERK) has also been shown to be one of the mitogen-activated protein kinases (MAPKs) activated by calcium influx [19]. It has been reported that ERK phosphorylation (pERK) in Vc and C1/C2 neurons occurs within 5min following orofacial noxious stimulation [20], [21]. In addition, pERK-immunoreactive (IR) cells have been reported to be organized somatotopically in Vc and C1/C2 following capsaicin injection into various orofacial regions, and ERK phosphorylation in these neurons increases as the stimulus intensity increases, indicating that ERK phosphorylation could be a reliable maker of neurons activated by a variety of noxious stimuli [22]. It has also been reported that Fos expression occurs in Vc and C1/C2 at 30 min following orofacial capsaicin injection and peaked at 120 min after that [20].

Therefore, Vi/Vc, Vc and C1/C2 could be target regions to study the modulation of nociceptive neurons following orofacial inflammation. To evaluate the functional role of GluR2 and GluR3 subunit in orofacial inflammatory pain, we analyzed nocifensive behavior, pERK and Fos expression in mouse Vi/Vc, Vc and C1/C2, and also Vc neuronal activity to address the hypothesis that trafficking of GluR2 and GluR3 subunits plays an important role in Vi/Vc, Vc and C1/C2 neuronal activity associated with orofacial inflammation using GluR2 delta7 knock-in (KI) and GluR3 delta7 KI mice. For secure inhibition of protein interaction, we deleted C-terminal seven amino acids including PDZ ligand sequence by introducing premature artificial stop codon into GluR2/3 genomic DNA regions.

## Results

### Nocifensive behavior

**Figure 1:**
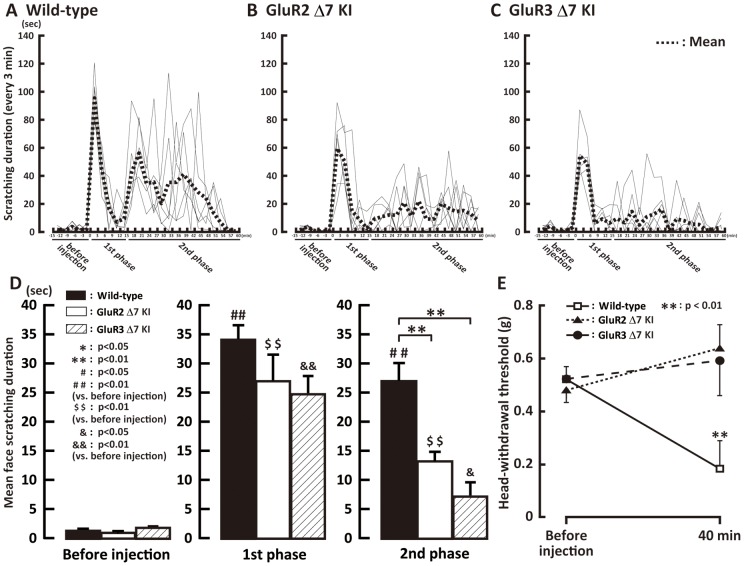
Face scratching and mechanical head-withdrawal threshold following subcutaneous formalin injection into the whisker pad. A, B and C: The duration of face scratching events in wild-type, GluR2 delta7 KI and GluR3 delta7 KI mice. Gray lines in A, B and C represent individual animal values and dotted lines indicate mean values. D: Mean face scratching duration after formalin injection was plotted before, and in the 1^st^ phase (0–15 min) and 2^nd^ phase (16–60 min) after formalin injection. E: Mean head-withdrawal threshold before and 40 min after formalin injection into the whisker pad skin in in the wild-type, GluR2 delta7 KI and GluR3 delta7 KI mice.

The duration of facial scratching was measured for 60 min after subcutaneous formalin injection into the whisker pad skin. The duration of face scratching events in individual mouse is shown in Fig. 1A, B and C. We divided the duration of scratches into 2 blocks after subcutaneous formalin injection: the 1^st^ phase (0–15 min) and the 2^nd^ phase (16–60 min) (Fig. 1D). Prior to formalin injection, mice scratched their facial skin for less than 5 s during the 3 min measurement period in wild-type, GluR2 delta7 KI and GluR3 delta7 KI mice. After subcutaneous formalin injection, scratching duration significantly increased in wild-type, GluR2 delta7 KI and GluR3 delta7 KI mice in the 1^st^ and 2^nd^ phases. The scratching duration in the 1^st^ phase was not different between wild-type, GluR2 delta7 KI and GluR3 delta7 KI mice in formalin-injected mice, but in the 2^nd^ phase, scratching duration was significantly shorter in GluR2 delta7 KI and GluR3 delta7 KI mice compared with that of wild-type mice (wild-type vs. GluR2 delta7 KI or GluR3 delta7 KI: p<0.01, n = 5).

We also measured the head-withdrawal threshold to mechanical stimulation of the whisker pad skin ipsilateral to the formalin injection in wild-type, GluR2 delta7 KI and Glu3 delta7 KI mice. The head-withdrawal threshold to mechanical stimulation of the whisker pad skin was significantly decreased at 40 min after formalin injection in the wild-type mice, while mechanical head-withdrawal threshold did not change in either GluR2 delta7 KI or GluR3 delta7 KI mice 40 min after formalin injection (Fig. 1E).

### pERK-IR cells in Vi/Vc, Vc and C1/C2

**Figure 2:**
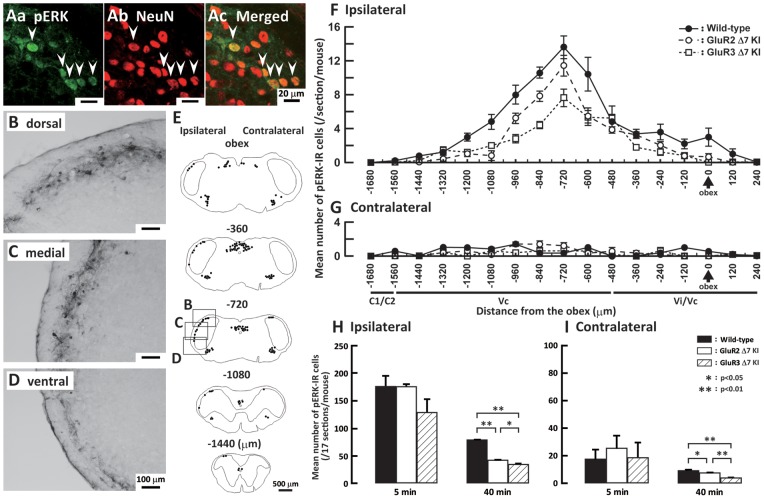
Expression of pERK-IR cells in Vi/Vc, Vc and C1/C2 after formalin injection. A: High magnification photomicrographs of pERK-IR cells (Aa), NeuN positive cells (Ab) and NeuN-labeled pERK-IR cells (Ac) at 5 min after formalin injection in the Vc of wild-type mice. B, C and D: low magnification photomicrographs of pERK-IR cells in the dorsal (B), middle (C) and ventral (D) portions of the Vc 5 min after formalin injection in wild-type mice. E: Camera lucida drawings of pERK-IR cells in the wild-type mice at 5 min after subcutaneous formalin injection into the whisker pad skin in sections from −1440 µm to the obex. F and G: Rostro-caudal distribution of pERK-IR cells in the wild-type, GluR2 delta7 KI and GluR3 delta7 KI mice 40 min after formalin injection in the ipsilateral and contralateral whisker pads, respectively. The mean number of pERK-IR cells in the ipsilateral (H) and contralateral (I) Vi/Vc, Vc and C1/C2 in the wild-type, GluR2 delta7 KI and GluR3 delta7 KI mice 40 min after subcutaneous formalin injection into the whisker pad skin. Boxes in the 3^rd^ panel in E indicate areas in B, C and D. Five min data are presented besides the 40 min ones in H and I.

The pERK-IR cells were expressed in Vi/Vc, Vc and C1/C2 following subcutaneous formalin injection into the whisker pad skin in wild-type, GluR2 delta7 KI and GluR3 delta7 KI mice. As illustrated in Fig. 2 Aa, Ab and Ac, all pERK-IR cells showed NeuN immunoreactivity indicating that all pERK-IR cells were defined as neurons. The pERK-IR cells were mainly expressed in the superficial laminae of the ipsilateral Vi/Vc, Vc and C1/C2 at 5 min after formalin injection (Fig. 2B, C, D, E, F and H), and a small number of pERK-IR cells was expressed in Vi/Vc, Vc and C1/C2 contralateral to the formalin injection (Fig. 2E, G and I). The largest number of pERK-IR cells was observed in the ipsilateral Vi/Vc and Vc at 600–720 µm caudal to the obex in wild-type, GluR2 delta7 KI and GluR3 delta7 KI mice (Fig. 2F). There was no significant difference in the number of pERK-IR cells on both sides of Vi/Vc, Vc and C1/C2 at 5 min after subcutaneous formalin injection into the whisker pad skin (Fig. 2H and I). On the other hand, the number of pERK-IR cells on both sides of Vi/Vc, Vc and C1/C2 was significantly larger in wild-type mice compared with that of GluR2 delta7 KI and GluR3 delta7 KI mice at 40 min after formalin injection (Fig. 2H and I). Since some previous studies have documented that ERK phosphorylation occurs in glial cells in rats with sciatic nerve injury [23], [24], we also tested if pERK-IR cells expressed glial fibrillary acid protein (GFAP) and ionized calcium-binding adaptor molecule-1 (Iba1) that are markers for astroglial and microglial cells, respectively. Many Iba1-IR cells (Figure S1C and D) and few GFAP-IR cells (Figure S1A and B) were observed in Vc of formalin-injected mice. However, pERK-IR cells did not show GFAP and Iba1 immunoreactivities in formalin–injected mice, but all pERK-IR cells showed NeuN immunoreactivity.

We also studied the ERK phosphorylation in the Vi/Vc, Vc and C1/C2 neurons following subcutaneous capsaicin injection into the whisker pad skin. We could not observe any significant difference in the number of pERK-IR cells between GluR2 or GluR3 delta7KI mice and wild type mice 5 (Figure S2A) and 40 min (Figure S2B) after capsaicin injection.

### Fos protein-IR cells in Vi/Vc, Vc and C1/C2

**Figure 3:**
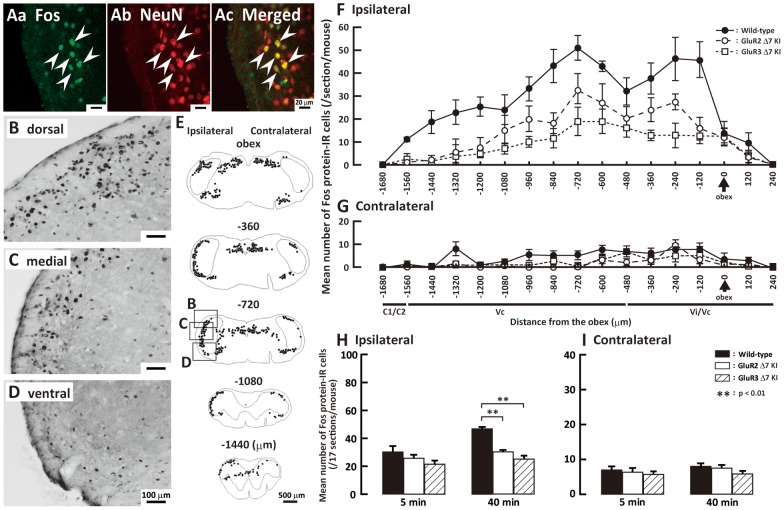
Expression of Fos protein-IR cells in Vi/Vc, Vc and C1/C2 after formalin injection. A: High magnification photomicrographs of Fos protein-IR cells (Aa), NeuN positive cells (Ab) and NeuN-labeled pERK-IR cells (Ac) at 40 min after formalin injection in the Vc of wild-type mice. B, C and D: low magnification photomicrographs of Fos protein-IR cells in the dorsal (B), middle (C) and ventral (D) portions of the Vc 40 min after formalin injection in wild-type mice. E: Camera lucida drawings of Fos protein-IR cells in the wild-type mice at 40 min after subcutaneous formalin injection into the whisker pad skin in sections from −1440 µm to the obex. Rostro-caudal distribution of Fos protein-IR cells at 40 min after formalin injection (F: ipsilateral side to injection, G: contralateral side to injection). The mean number of Fos protein-IR cells in the ipsilateral (H) and contralateral (I) Vi/Vc, Vc and C1/C2 in wild-type and GluR2 delta7 KI and GluR3 delta7 KI mice after subcutaneous formalin injection into the whisker pad skin. Boxes in the 3^rd^ panel in E indicate areas in B, C and D. Both 5 and 40 min data are presented in H and I.

Next, we analyzed Fos expression in Vi/Vc, Vc and C1/C2 neurons after subcutaneous formalin injection into the whisker pad skin. All Fos protein-IR cells expressed in Vc at 40 min after formalin injection showed NeuN immunoreactivity in wild-type mice indicating that all Fos protein-IR cells were defined as neurons (Fig. 3Aa, Ab and Ac). Many Fos protein-IR cells were observed in the superficial laminae and some in the deep laminae of Vi/Vc, Vc and C1/C2 at 40 min after formalin injection in wild-type, GluR2 delta7 KI and GluR3 delta7 KI mice (Fig. 3B, C, D, E, F and H). Fos protein-IR cells were expressed in Vi/Vc, Vc and C1/C2 with two peaks 120–240 µm and 720 µm caudal to the obex in wild-type and GluR2 delta7 KI mice as illustrated in Fig. 3E and F. The number of Fos protein-IR cells in the ipsilateral Vi/Vc, Vc and C1/C2 was significantly larger in wild-type mice compared to GluR2 delta7 KI and GluR3 delta7 KI mice at 40 min but not 5 min after subcutaneous formalin injection in to the whisker pad skin (Fig. 3H). Only a small number of Fos protein-IR cells was observed in the contralateral Vi/Vc, Vc and C1/C2 after formalin injection, and no significant difference in the number of Fos protein-IR cells could be observed in the side contralateral to the formalin injection between wild-type mice, GluR2 delta7 KI and GluR3 delta7 KI mice (Fig. 3I).

### Effect of AMPAR or NMDAR antagonist on pERK-IR or Fos protein-IR

**Figure 4:**
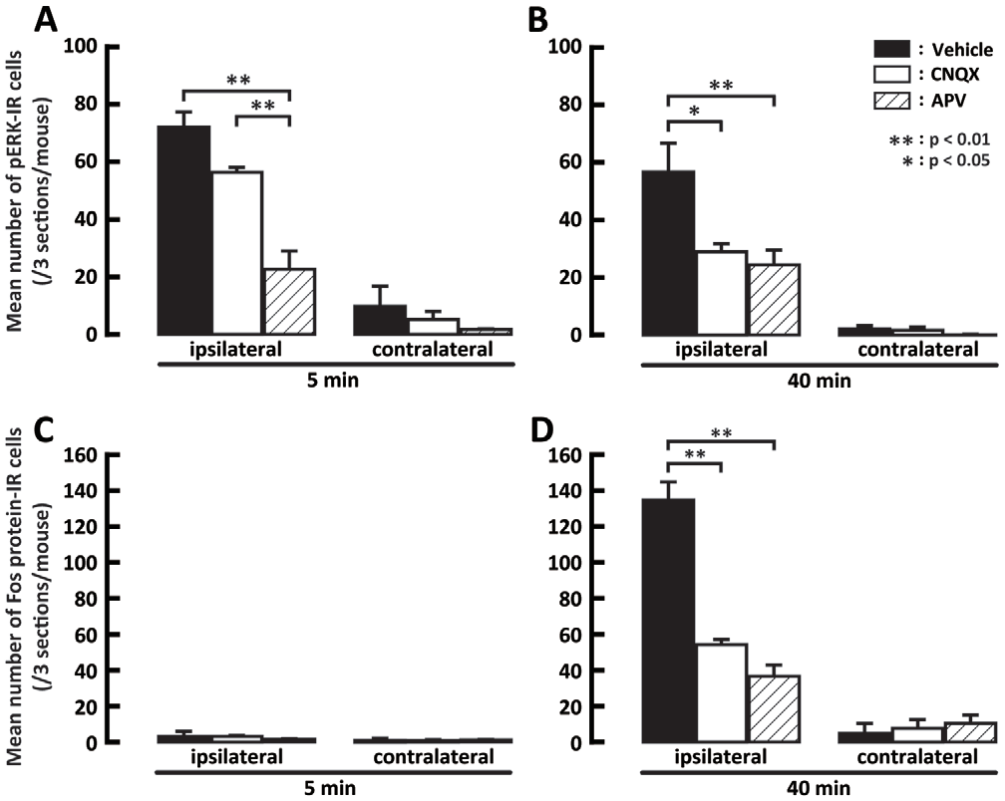
Effect of intrathecal administration of vehicle, CNQX or APV on pERK or Fos expression. A and B: Mean number of pERK-IR cells in ipsilateral and contralateral Vi/Vc, Vc and C1/C2 regions following i.t. administration of vehicle, CNQX or APV 5 min (A) and 40 min (B) after subcutaneous formalin injection into the whisker pad skin in wild-type mice. C and D: Mean number of Fos protein-IR cells in ipsilateral and contralateral Vi/Vc, Vc and C1/C2 regions following i.t. administration of vehicle, CNQX or APV 5 min (C) and 40 min (D) after subcutaneous formalin injection into the whisker pad skin in wild-type mice.

Since there are no reports on whether the AMPA receptor is involved in ERK phosphorylation in Vi/Vc, Vc and C1/C2 neurons, we studied the effect of CNQX in comparison with the effect of APV on ERK phosphorylation in this model to clarify if the AMAPA receptor is involved in ERK phosphorylation and Fos expression in the formalin-injected wild-type mice. The mechanism underlying inflammatory pain in our model may be similar to the mechanistic concept reported by Park et al. in which the NMDA receptor is involved in GluR2 receptor internalization following peripheral inflammations, but we cannot delineate the mechanisms underlying orofacial persistent inflammatory pain since we did not specifically test for NMDA receptor involvement in the GluR2 delta7 KI and GluR3 delta7 KI mice. The number of pERK-IR cells was significantly smaller in APV-injected wild-type mice than that of vehicle- and CNQX-injected wild-type mice at 5 min after subcutaneous formalin injection into the whisker pad skin (Fig. 4A). At 40 min after formalin injection, the number of pERK-IR cells was significantly smaller in CNQX- or APV-injected wild-type mice compared with vehicle-injected wild-type mice (Fig. 4B). The number of Fos protein-IR cells at 40 min but not 5 min after formalin injection was also significantly smaller in CNQX- or APV-injected wild-type mice than in vehicle-injected wild-type mice (Fig. 4C and D). The number of Fos protein-IR cells was also significantly smaller in APV-injected wild-type mice than in CNQX-injected wild-type mice (Fig. 4D).

### Vc neuronal activities

**Figure 5:**
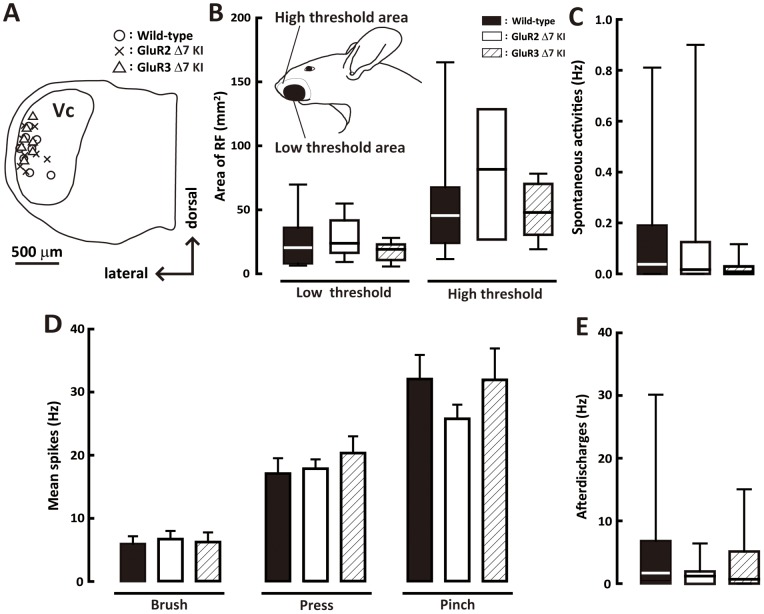
Reponses of WDR neurons in the Vc. A: recording sites of WDR neurons in the Vc of the wild-type, GluR2 delta7 KI and GluR3 delta7 KI mice. B: Low- and high-threshold RF areas of WDR neurons in Vc. C: Spontaneous activities of WDR neurons in the Vc. D: Mean spikes of WDR neurons in the Vc following graded mechanical stimulation (brush, press and pinch) of the whisker pad skin. E: Mean afterdischarges of WDR neurons in the Vc followed by noxious mechanical stimulation. The RF size was presented only for untreated animals since spontaneous activity was too high to allow for record accurate delineation of RF size after formalin injection in C.

Since the largest number of pERK-IR and Fos protein-IR cells could be observed in the Vc, we analyzed the Vc neuronal activities in wild-type, GluR2 delta7 KI mice and GluR3 delta7 KI mice using extracellular recording techniques. Wide dynamic range (WDR) neurons (wild-type: n = 12, GluR2 delta7 KI: n = 13, GluR3 delta7 KI: n = 10) and nociceptive specific (NS) neurons (wild-type: n = 5, GluR2 delta7 KI: n = 1, GluR3 delta7 KI: n = 5) were recorded from the Vc, and mechanical evoked responses, spontaneous of activities and afterdischarges of these neurons were analyzed in wild-type mice, GluR2 delta7 KI mice or GluR3 delta7 KI mice. Since we only had a limited sample of NS neurons, only the response properties of WDR neurons were analyzed in this study. The RFs of WDR neurons analyzed in this study were located in the whisker pad skin. WDR neurons were located both in the deep and superficial laminae in the Vc (Fig. 5A). Baseline RF size, mean spike frequency to mechanical stimulation of the whisker pad skin and spontaneous activity of WDR neurons were not significantly different between wild-type, GluR2 delta7 KI and GluR3 delta7 KI mice (Fig. 5B, C, D and E).

**Figure 6:**
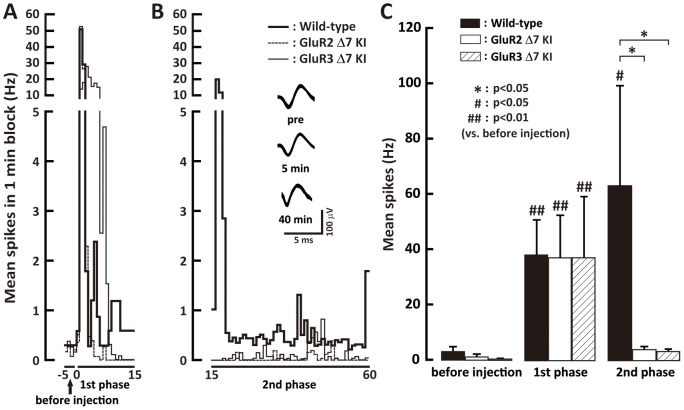
Responses of Vc WDR neurons following subcutaneous formalin injection into the whisker pad. Post-stimulus time histograms at the 1^st^ phase (A) and at the 2^nd^ phase (B). C: Mean firing frequency of Vc WDR neurons after subcutaneous formalin injection into the whisker pad skin before injection, at 1^st^ phase and 2^nd^ phases after subcutaneous formalin injection into the whisker pad skin.

After formalin injection, the spontaneous activity of Vc WDR neurons in wild-type, GluR2 delta7 KI and GluR3 delta7 KI mice showed initially rapid increase in firing frequency, then was decreased at 10 min, and increased again 15 min later (Fig. 6A and B). The firing frequency of the spontaneous activity in wild-type, GluR2 delta7 KI and GluR3 delta7 KI mice was significantly higher in the 1^st^ phase after subcutaneous formalin injection into the whisker pad skin compared to that before formalin injection (Fig. 6C). We did not observe any significant difference in firing frequency of Vc WDR neurons in the 1^st^ phase between these 3 types of mice after the formalin injection (Fig. 6C). In the 2^nd^ phase, spontaneous activity after formalin injection was significantly higher in wild-type mice than that of GluR2 delta7 KI and GluR3 delta7 KI mice, no difference in spontaneous activity could be observed between GluR2 delta7 KI and GluR3 delta7 KI mice (Fig. 6C).

## Discussion

In order to assess our hypothesis that GluR2 and GluR3 trafficking is involved in persistent inflammatory pain in the orofacial region, we used an inflammatory pain model (the formalin test) to study nocifensive behavior, pERK and Fos expression in GluR2 and GluR3 d7 KI mice. The number of pERK-IR cells was significantly decreased bilaterally in the Vi/Vc, Vc and C1/C2 in GluR2 delta7 KI and GluR3 delta7 KI mice compared to wild-type mice in the 2^nd^ phase, and was also significantly smaller in GluR3 delta7 KI compared to GluR2 delta7 KI mice. The number of Fos protein-IR cells in the ipsilateral Vi/Vc, Vc and C1/C2 was also significantly smaller in GluR2 delta7 KI and GluR3 delta7 KI mice. WDR neurons showed significantly lower spontaneous activity in GluR2 delta7 KI and GluR3 delta7 KI mice than wild-type mice after formalin injection. These findings suggest that GluR2 and GluR3 trafficking is similarly involved in the enhancement of Vi/Vc, Vc and C1/C2 WDR neuronal excitability associated with enhanced face scratching in the 2^nd^ phase of the formalin test, and also suggest that GluR3 trafficking may be involved in inflammatory pain as well as other sensory functions via the intracellular ERK cascade.

### Involvement of GluR2 and GluR3 trafficking in orofacial inflammatory pain

We observed that duration of face scratching was significantly longer in wild-type mice compared with GluR2 delta7 KI and GluR3 delta7 KI mice in the 2^nd^ phase of the formalin test, whereas there was no difference between these 3 types of mice in the 1^st^ phase. Moreover, the head-withdrawal threshold to mechanical stimulation of the whisker pad skin was significantly lower in wild-type mice at 40 min after formalin injection, while no change in head-withdrawal threshold could be observed in GluR2 delta7 KI and GluR3 delta7 KI mice following formalin injection. Furthermore, it has been reported that NMDA receptor-mediated triggering of protein kinase C (PKC) activation is required for the induction and maintenance of CFA-induced dorsal horn GluR2 internalization which is involved in hyperalgesia following CFA injection into the hind paw [5]. We could not also observe a significant difference in the face scratching duration between GluR2 delta7 KI and GluR3 delta7 KI mice following formalin injection. Together with previous data [5], our results suggest that trafficking of GluR2 and GluR3 is similarly involved in the occurrence of the orofacial hyperalgesia as well as the allodynia that occurs in orofacial inflammatory pain conditions.

### Effect of GluR2 delta7 KI and GluR3 delta7 KI on pERK and Fos expression

Based on some previous studies, the present study utilized the phosphorylation of ERK as a marker of the acute activation of nociceptive neurons [16], [20], [21], [29]. We observed pERK-IR cells in the Vc within 5 min after subcutaneous formalin injection into the whisker pad skin. Furthermore, few Fos protein-IR cells were expressed at 5 min but a large number of them were expressed at 40 min in the Vc after subcutaneous formalin injection into the whisker pad skin. These findings indicate that ERK phosphorylation and Fos expression have different time courses following strong noxious stimulation, and further suggest that pERK and Fos are differentially involved in the processing of nociceptive information in the trigeminal region.

ERK is known to be phosphorylated 5–10 min after acute noxious stimuli such as capsaicin application or noxious heating of the face or intraoral structures [16], [20], [21], [25]. On the other hand, we observed pERK-IR cell expression at 40 min after formalin injection in the wild-type mice as well as at 5 min, whereas Fos expression peaked at 60–120 min after noxious stimulation. The time course difference in ERK phosphorylation between previous findings and the present data may be related to the different stimuli used. Formalin may cause a longer-lasting injury of tissues compared to capsaicin or noxious heating, which may result in a longer-lasting phosphorylation of ERK in formalin-injected mice compared to capsaicin-injected mice. Furthermore, we could not observe any significant differences in the number of pERK-IR cells 5 and 40 min following subcutaneous capsaicin injection into the whisker pad skin between wild-type mice and GluR2 delta7 KI or GluR3 delta7 KI mice. This suggest that formalin and capsaicin cause different effect on GluR2 or GluR3 trafficking in the Vi/Vc, Vc and C1/C2 neurons.

To clarify whether AMPA or NMDA receptors in Vi/Vc, Vc and C1/C2 neurons are involved in ERK phosphorylation after subcutaneous formalin injection into the whisker pad skin, the effects of i.t. administration of the AMPA receptor antagonist CNQX and NMDA receptor antagonist APV on ERK phosphorylation and Fos expression are studied in formalin-injected rats. We observed a significant reduction of ERK phosphorylation and Fos expression at 40 min after formalin injection in CNQX- and APV-injected mice and also a significant reduction of ERK phosphorylation at 5 min after formalin injection in APV-injected mice compared with vehicle-injected mice, suggesting that both AMPA and NMDA receptors are involved in the enhancement of neuronal excitability at 40 min after formalin injection, and that NMDA receptors are also involved in the enhancement of the neuronal excitability at 5 min after formalin injection.

We also observed a difference in the magnitude of ERK phosphorylation in Vi/Vc, Vc and C1/C2 neurons between GluR2 delta7 KI and GluR3 delta7 KI mice following subcutaneous formalin injection into the whisker pad skin. Ca^2+^ permeability is well known to be different between GluR2 and GluR3 in a variety of brain regions [26]. The distribution pattern of GluR2 and GluR3 subunits also is reported to be different in the spinal DH, with GluR2 mainly distributed in lamina II, whereas GluR3 predominates in laminae I and III [9]. These may affect the excitability of Vi/Vc, Vc and C1/C2 neurons and account for the difference in ERK phosphorylation in Vi/Vc, Vc and C1/C2 neurons between GluR2 delta7 KI and GluR3 delta7 KI mice.

Since some previous studies have documented that ERK phosphorylation occurs in glial cells in rats with sciatic nerve injury [23], [24], we also tested if pERK-IR cells expressed GFAP and Iba1 that are markers for astroglial and microglial cells, respectively. There was no evidence of any pERK-IR cells showing GFAP and Iba1 immunoreactivities in formalin-injected mice, but all pERK-IR cells showed NeuN immunoreactivity. These findings suggest that ERK phosphorylation may occur in neurons but not in astroglial or microglial cells in Vc in this model.

### Functional differences of GluR2 and GluR3 trafficking in orofacial inflammatory pain

Mechanical-evoked responses and spontaneous activity of Vc WDR neurons were analyzed in wild-type, GluR2 delta7 KI and GluR3 delta7 KI mice before formalin injection, but no differences in these Vc neuronal properties could be observed. It is likely that GluR2 and GluR3 in the cell membrane may be functionally similar between GluR2 delta7 KI mice, GluR3 delta7 KI mice and wild-type mice in the normal state. Although significantly higher spontaneous activity in functionally identified Vc WDR neurons was documented in wild-type and GluR2 delta7 KI and Glu3 delta7 KI mice compared with that of vehicle-injected mice in the 1^st^ phase after formalin injection, no differences in firing frequency could be observed in these 3 types of mice in this phase. On the other hand, spontaneous activity was significantly higher in formalin-injected wild-type mice but not formalin-injected GluR2 delta7 KI and GluR3 delta7 KI mice compared with that of vehicle-injected mice in the 2^nd^ phase, and also spontaneous activity was not significantly different between formalin-injected GluR2 delta7 KI and Glu3 delta7 KI mice. These observations suggest that the trafficking of GluR2 and GluR3 subtypes in the cell membrane of Vc neurons have important roles in the enhancement of spontaneous neuronal excitability of Vc WDR neurons following facial inflammation. The deficit of the membrane trafficking of GluR2 and GluR3 may not cause alteration of AMPAR expression in the cell membrane, resulting in the attenuation of increased spontaneous activity following formalin injection.

### Conclusions

We observed the significant difference in ERK phosphorylation in Vi/Vc, Vc and C1/C2 neurons between GluR2 delta7 KI and GluR3 delta7 KI mice in the 2^nd^ phase of the formalin test applied to the mouse whisker pad. Otherwise, nocifensive behavior, Fos expression and WDR spontaneous activity in whisker pad-inflamed mice were similar in GluR2 delta7 KI and GluR3 delta7 KI mice. The present findings suggest that the membrane trafficking of GluR2 and GluR3 subtypes is similarly involved in enhancement of Vi/Vc, Vc and C1/C2 neuronal excitability in the 2^nd^ phase of the formalin-induced hyperalgesia, and also suggest that the GluR3 trafficking may be involved in inflammatory pain as well as other sensory functions via the intracellular ERK cascade. The use of specific blockers of GluR2 or GluR3 trafficking would be helpful in future studies in order to clarify further the functional differences between these 2 types of AMPA receptor subtypes associated with orofacial persistent inflammatory pain.

## Materials and Methods

### Animals

Adult male C57BL/6, wild-type (n = 116) and GluR2 delta7 KI (n = 50) and GluR3 delta7 KI mice (n = 54) (30–35 g) were used in the present study. These mutant mice were viable and showed normal appearances. Since GluR3 is located on the X-chromosome, male mice carrying one mutant allele were grouped into homozygous mutants as well as female homozygous mice.

The mice were housed under 12 h light/dark cycle conditions and had free access to food and water except during the test period. To minimize animal suffering, the number of animals used was based on the minimum required for statistically valid results. Behavioral and electrophysiological analysis using wild-type and mutant mice (GluR2 delta7 KI and GluR3 delta7 KI mice) were carried out in a blind manner. All methods and experimental approaches were approved by the Animal Care Committee of Nihon University and by the Committee for DNA transformation of Nihon University.

### PCR-based genotyping

Genotype in each generation of mutant mice was confirmed by PCR analysis on isolated genomic DNA as previously described [27]. Briefly, tail samples from mice were digested with 0.5 mg/ml proteinase K at 55°C overnight. Following phenol extraction, chloroform extraction and precipitation with ethanol, genomic DNA was suspended with tetra EDTA (TE) buffer. PCR was performed on the isolated genomic DNA using taq DNA polymerase (TaKaRa Ex Taq^TM^, Otsu, Japan) and primer sets (primer #21–23 for GluR2 D7: #21, 5′-ACA GAG GAA GGT AGT GGA AGG GAG-3′; #22, 5′-CTT GGT TTG GTT GTT GGT CAT AGC-3′; #23, 5′-CTA GTG AAC CTC TTC GAG GGA C-3′; primer #31-33 for GluR3 D7: #31, 5′-CCA ATA CTC CAC AGG GGC AAT TTA TC-3′; #32, 5′-CCG TTG ACT GTT TTG AAT CTC ACA CC-3′; #33, 5′-CTA GTG AAC CTC TTC GAG GGA C-3′). Size of the amplification product was estimated by gel electrophoresis for genotyping (350 bp for wild-type and 210 bp for GluR2; 440 bp for wild-type and 300 bp for GluR3).

### Nocifensive behavior

A total of 33 mice were used for the behavioral experiments (formalin: n = 18, mechanical: n = 15). Formalin solutions were prepared from commercially available stock formalin further diluted in isotonic saline to 4%. Before injection, mice were placed in a test box (29×29×34.5 cm) for 15 min and scratching duration was recorded every 3 min as baseline, and the mean of the baseline scratching duration was calculated from 5 counts. Formalin solution (10 µl) was injected subcutaneously with a 27-gauge needle attached to Hamilton syringe into the center of the left whisker pad skin, with only minimal animal restraint. Injected animals were immediately placed back into the test box for a 60-min observation period. A nociceptive behavior score was determined by measuring the mean face scratching duration (sec) every 3 min in each animal following formalin injection [28]. Analysis of the behavior was made by an investigator who was blinded to the animal's group assignment. The head-withdrawal threshold to mechanical stimulation of the whisker pad skin by von Frey filament was evaluated 15 min before injection and 40 min after the injection in the side ipsilateral to the formalin injection.

### Immunohistochemistry

Mice were transcardially perfused with 1% paraformaldehyde (PFA) (50 ml) followed by 4% PFA in 0.1 M phosphate buffer (pH 7.4, 50 ml) at 5 or 40 min after subcutaneous formalin injection or capsaicin into the whisker pad skin (n = 15 in each time point). The bilateral medulla and C1/C2 were removed and post-fixed with 4% PFA for 3 days at 4°C. The tissues were then transferred to 20% sucrose (w/v) in phosphate-buffered saline (PBS) overnight for cryoprotection. Thirty µm thick sections were cut from the brain stem and collected in PBS. Free-floating tissue sections were rinsed in PBS, and then incubated with 6% H_2_O_2_ in PBS for 30 min after washing with 10% normal goat serum (NGS) in PBS for 2 h. The sections were reacted with rabbit anti-Phospho-p44/42 MAP kinase (Thr202/Tyr204) antibody (1: 1000; Cell Signaling Technology, Danvers, MA) for 72 h at 4°C. The antibodies used in this study were all commercially available. Next, these sections were incubated with biotinylated goat anti-rabbit IgG (1 : 600; Vector Labs, Burlingame, CA) for 2 h at room temperature. After rinsing with PBS, the sections were incubated with peroxidase-conjugated avidin-biotin complex (1 : 100; Vector Labs) for 2 h at room temperature. After rinsing with 0.05 M Tris Buffer (TB), the sections were incubated in 0.035% 3,3′-diaminobenzidine-tetra HCl (Sigma, St. Louis, MO), 0.2% nickel ammonium sulfate, and 0.05% peroxide in 0.05 M TB (pH 7.4). After rinsing with PBS, the sections were serially mounted on gelatin-coated slides, dehydrated and cover-slipped. The pERK-immunoreactive (IR) cells Vi/Vc, Vc and C1/C2 were drawn under a light microscope equipped with camera lucida (Neurolucida 2000, MicroBrightField, Colchester, UT). Further, at 5 or 40 min after subcutaneous formalin injection into the whisker pad skin, the medulla and C1/C2 were removed, post-fixed and cryoprotected, and free-floating tissue sections were cut as above (n = 15 in each time point). After rinsing with PBS, 3% NGS in PBS for 2 h, the sections were incubated for 24 h with rabbit anti-c-fos antibody (1 : 20,000; c-fos ab-5, Darmstadt, Germany) in 3% NGS at 4°C. Other immunohistochemical staining was performed as described above in pERK immunohistochemistry.

The obex level was defined as at section before the appearance of the central canals as illustrated in Fig. 2E and Fig 3E. The Vi/Vc region, Vc region and C1/C2 region were defined as those sections from +240 to −360 µm to the obex, from −480 to −1440 µm to the obex and from −1560 to −1680 µm to the obex in mice. Every 120 µm sections were collected, and all pERK-IR and Fos protein-IR cells distributed within Vi/Vc, Vc and C1/C2 were drawn under Neurolucida 2000. The number of pERK-IR cells or Fos protein-IR cells was counted within these three nuclei (Vi/Vc, Vc and C1/C2) in the ipsilateral and contralateral side to formalin injection. The mean number of pERK-IR or Fos protein-IR cells was calculated from the total number of IR cells in 17 sections from −1680 µm to +240 µm to the obex in each mouse.

Double immunofluorescence histochemistry was also carried out to determine if pERK and Fos are expressed in neurons (n = 5). Five and 40 min after formalin injection, wild-type mice were perfused, the whole brain including medulla and upper cervical spinal cord was removed and post-fixed in the same fixative for 3 days at 4°C (5 min for pERK staining, 40 min for Fos staining). Free-floating tissue sections (30 µm) were rinsed in PBS, 10% NGS in PBS for 2 h, and then reacted with rabbit anti-Phospho-p44/42 MAPK antibody (1 : 300; Cell Signaling Technology) or rabbit anti-c-fos antibody (1 : 1,000; c-fos ab-5, Oncogene, MA) and mouse anti-NeuN antibody (1 : 1000; Chemicon, Temecula, CA) over night at 4°C and secondary antibodies (FITC- and rhodamine-conjugated, 1 : 100; Jackson ImmunoResearch, West Grove, PA) for 2 h at room temperature in a dark room. After rinsing with PBS, the sections were mounted on slides and cover slipped in PermaFluor (Sigma). Photographs were taken with a confocal laser microscope (LSM 510, Carl Zeiss, Oberkochen, Germany). To evaluate whether ERK was phosphorylated in the Vc after subcutaneous formalin injection into the whisker pad, GFAP and Iba1 immunoreactivities were also studied in formalin injected wild-type mice. For pERK/GFAP or pERK/Iba1 double immunofluorescence histochemistry, rabbit anti-GFAP (1∶1000; Dako Z0334) and rabbit anti-Iba1 (1∶2000, Wako) were used as primary antibodies instead of anti-NeuN antibody, and other immunohistochemical staining was performed as described above.

We assessed the effect of intrathecal (i.t.) administration of the AMPA receptor antagonist 6-yano-7-nitoquinoxa-line 2,3-dione (CNQX, Research Biochemicals International, Natic, MA) and the NMDA receptor antagonist D(-)-2-amono-5-phosphonopentanoic acid (APV, Research Biochemicals International) on pERK-IR cell and Fos protein-IR cell expression in Vi/Vc, Vc and C1/C2 were also analyzed in the formalin-injected wild-type mice (n = 60). Vehicle, CNQX (100 µM) or APV (50 µM) was applied to the medulla using a 30 gauge needle connected with p10 silicon tube. After exposure the dura mater covering medulla, the 30 gauge needle was inserted into the subdural space and its tip was placed dorsally in the obex level so as to prevent mechanical damage of the medulla. Six hours after the needle insertion, vehicle, CNQX or APV was injected. Five min after i.t. injection of vehicle, CNQX or APV, mice were perfused at 5 and 40 min after subcutaneous formalin injection into the whisker pad skin and processed for pERK and Fos immunohistochemistries (n = 5 each group).

### Single neuron recording

A total of 35 mice were used for single neuronal recording experiments (wild-type; n = 13, GluR2 delta7 KI; n = 9, GluR3 delta7 KI; n = 13). Mice were anesthetized with Urethane (1.5 g/kg, i.p.) and the trachea and left femoral vein were cannulated to allow artificial respiration and intravenous administration of drugs, respectively. Anesthesia was maintained with halothane (1%) mixed with oxygen during surgery. Mice were mounted securely in a stereotaxic frame with ear bars and nose holder. The dorsal neck skin was incised in the midline and the neck muscles over the brain stem were pushed aside. Then, the first cervical bone and dura membrane over the caudal part of the trigeminal spinal nucleus was removed. After surgery, anesthesia was maintained throughout the experiment by continuous inhalation of halothane (1%) mixed with oxygen. During the recording sessions, mice were immobilized with pancuronium bromide (1 mg/kg/h, i.v.) and ventilated artificially. Rectal temperature was maintained at 37–38°C by a thermostatically-controlled heating pad (ATB-1100, Nihon Koden, Japan). An electrocardiogram was also monitored and the heart rate was kept 360–460/min during the experiments. When the heart rate was increased following mechanical stimulation of the face, the concentration of halothane was increased to 2–3%.

The recording microelectrode was advanced at 1 µm steps in the Vc and single unit activities were searched by applying mechanical stimulations (pressure or brush) to the whisker pad skin region. When single unit activity was isolated, graded mechanical stimuli were delivered to the skin by brushing with a camel hair brush, pressing with a large arterial clip (FB426R, Aesculap, Germany) and pinching with a small arterial clip applied to the most sensitive area of the neuronal receptive fields (RFs) [29], [30]. Spontaneous activities of the neurons were first recorded for 60 s before applying mechanical stimulation. Neurons were then classified as low-threshold mechanosensitive (LTM) neurons, WDR neurons, or NS neurons based on their response properties to mechanical stimulation of the RF. To avoid sensitization by noxious stimulation, repeated noxious stimuli were not used to search for high-threshold mechanosensitive neurons. After classification of the neuron, 4% formalin was injected subcutaneously into the center of the neuronal RF. Spontaneous activities were recorded for 60 min after the injection. The waveforms of each neuron were amplified using a differential amplifier (high cut-off: 3 KHz, low cut-off: 30 Hz; Plexon Inc., Dallas, TX) and identified using Spike 2 software (CED, Cambridge, UK). Neuronal responses were saved on computer disk for subsequent off-line analysis of signals. Peristimulus time histograms (bin width  = 1 s) were generated in response to brush (1 Hz, 5 times), pressure (5 s) and pinch (5 s) and spike frequency was calculated. Afterdischarges were also calculated as the mean firing frequency for 10 s just after pinching of the center of the RFs.

It should however be noted that in the recording experiments, since enough number of nociceptive specific neurons could not be obtained for statistical analysis, only WDR neurons were analyzed, and spontaneous activity became too high to record accurately RF size after formalin injection. Thus, the RF size after formalin injection could not be analyzed.

### Statistical analysis

Behavioral data were obtained as individual and mean values, and were analyzed using analysis of variance (ANOVA) on ranks with the Newman-Keuls test. RF sizes, spontaneous activity and afterdischarges in the neuronal recording were obtained as median values, and were analyzed using Kruskal-Wallis test. All other data are presented as mean ± SEM for data from the extracellular recording experiments, whole cell recording and the immunohistochemical experiments and were analyzed using One-way ANOVA followed by the Tukey test. Two-way repeated measures ANOVA was used for the analysis of time-course changes in the numbers of pERK-IR cells and Fos protein-IR cells. Differences were considered as significant at p<0.05.

## Supporting Information

Figure S1 **Photomicrographs of ERK phosphorylation, GFAP and Iba1 in the Vc after subcutaneous formalin injection into the whisker pad in formalin–injected mice.** A, B and C: pERK-IR cells (A), GFAP-IR cells (B) and pERK-IR + GFAP-IR cells (C) 5 min after subcutaneous formalin injection into the whisker pad skin; D, E and F: pERK-IR cells (D), Iba1-IR cells (E) and pERK-IR + Iba1-IR cells (F) 5 min after subcutaneous formalin injection into the whisker pad skin; G, H and I: pERK-IR cells (G), pERK-IR cells (H) and pERK-IR + GFAP-IR cells (I) 40 min after subcutaneous formalin injection into the whisker pad skin; J, K and L: pERK-IR cells (J), pERK-IR cells (I) and pERK-IR + GFAP-IR cells (J) 40 min after subcutaneous formalin injection into the whisker pad skin.
(TIF)

Figure S2 **pERK-IR and Fos protein-IR cells in the Vi/Vc, Vc and C1/C2 following capsaicin injection into the whisker pad skin.** The number of pERK-IR in the Vi/Vc, Vc and C1/C2 of GluR2 or GluR3 Δ7KI mice and wild type mice 5 (A) and 40 min (B) after capsaicin injection into the whisker pad skin.
(TIF)
