# Supplementary figures and images for: Involvement of AMPA Receptor GluR2 and GluR3 Trafficking in Trigeminal Spinal Subnucleus Caudalis and C1/C2 Neurons in Acute-Facial Inflammatory Pain
Source: PLoS One. 2012 Aug 24;7(8):e44055. doi: 10.1371/journal.pone.0044055 (PMC3427165; doi:10.1371/journal.pone.0044055)

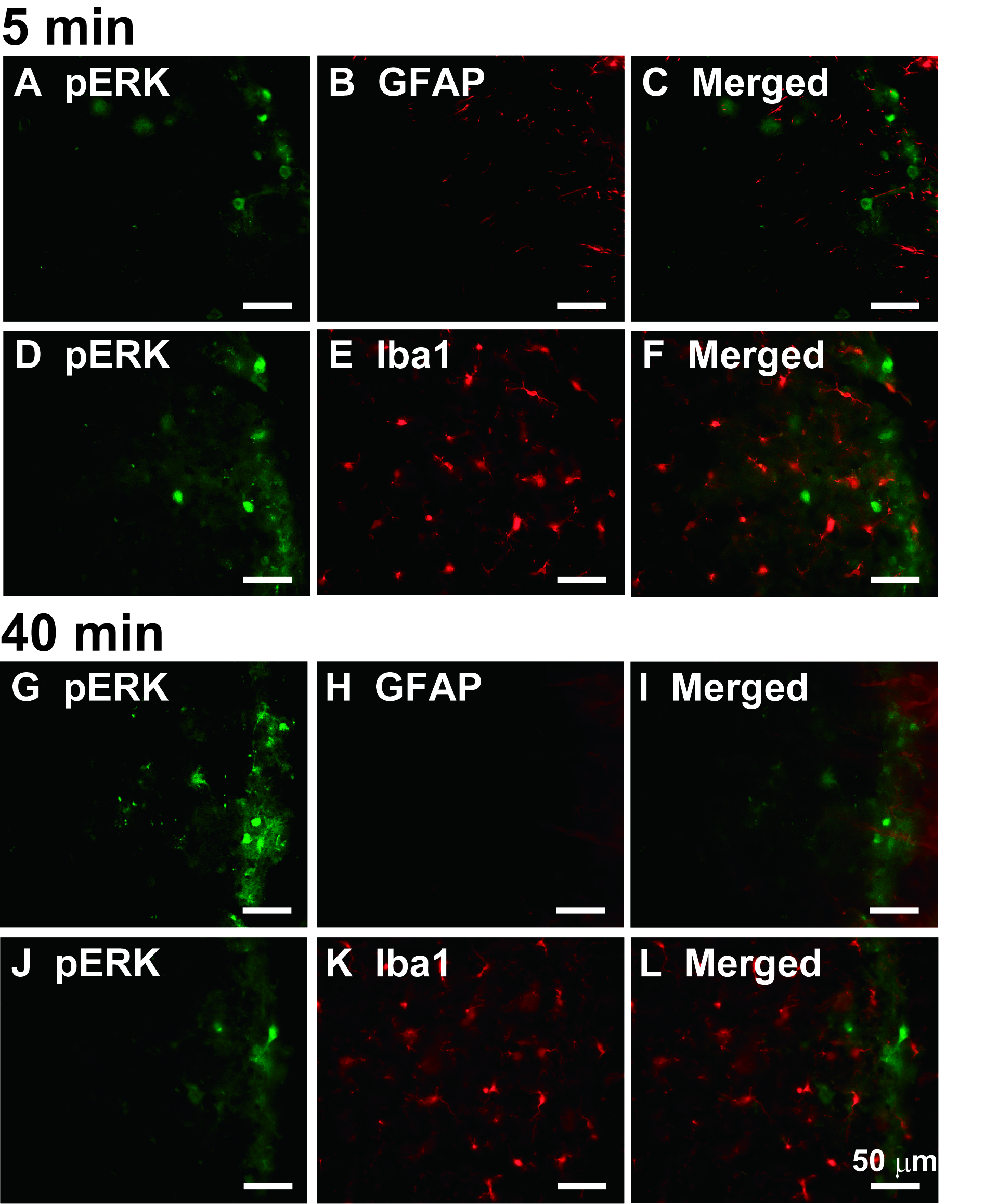

Supplement: Figure S1 — Photomicrographs of ERK phosphorylation, GFAP and Iba1 in the Vc after subcutaneous formalin injection into the whisker pad in formalin–injected mice. A, B and C: pERK-IR cells (A), GFAP-IR cells (B) and pERK-IR + GFAP-IR cells (C) 5 min after subcutaneous formalin injection into the whisker pad skin; D, E and F: pERK-IR cells (D), Iba1-IR cells (E) and pERK-IR + Iba1-IR cells (F) 5 min after subcutaneous formalin injection into the whisker pad skin; G, H and I: pERK-IR cells (G), pERK-IR cells (H) and pERK-IR + GFAP-IR cells (I) 40 min after subcutaneous formalin injection into the whisker pad skin; J, K and L: pERK-IR cells (J), pERK-IR cells (I) and pERK-IR + GFAP-IR cells (J) 40 min after subcutaneous formalin injection into the whisker pad skin. (TIF) [file pone.0044055.s001.tif]

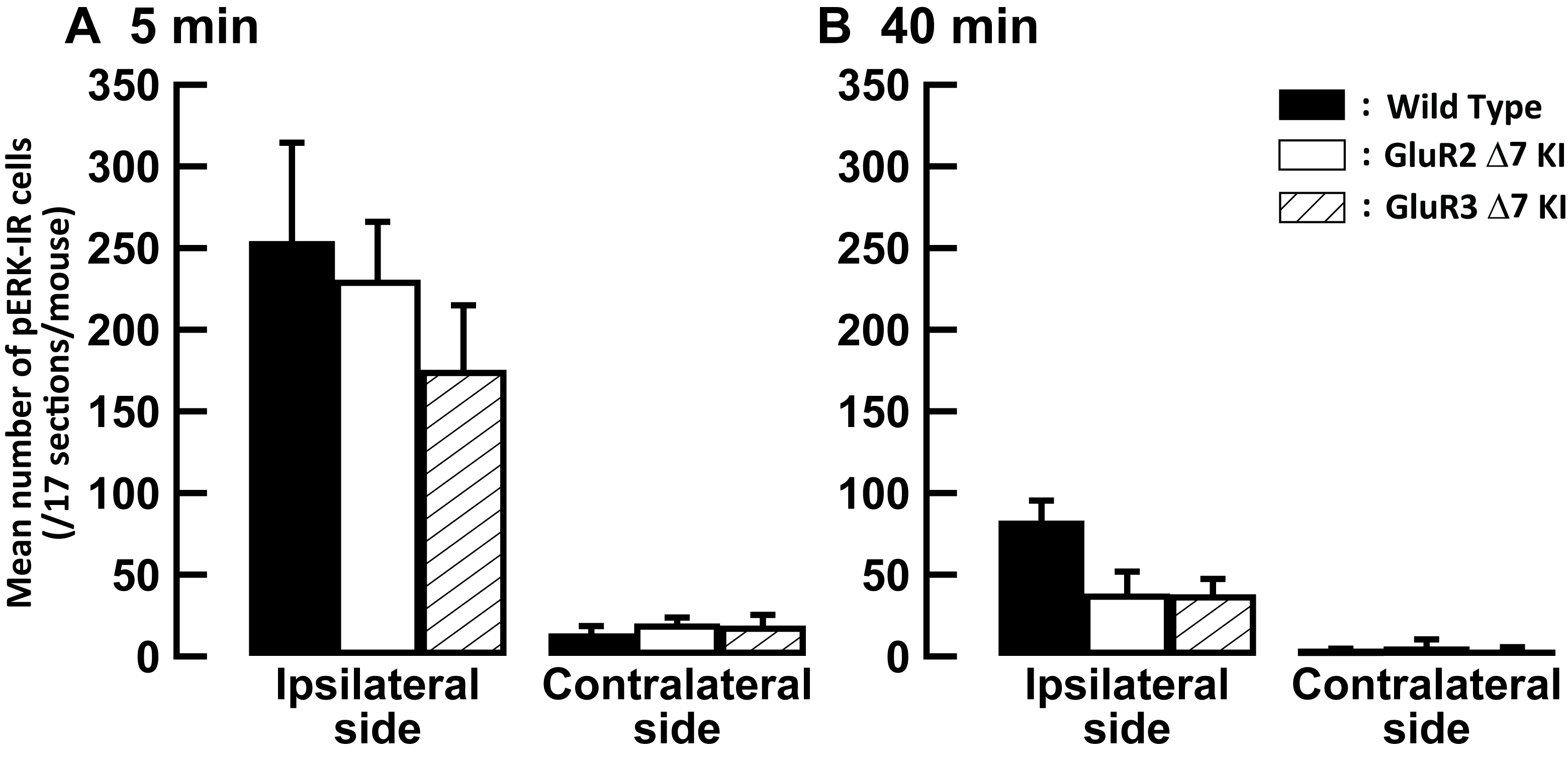

Supplement: Figure S2 — pERK-IR and Fos protein-IR cells in the Vi/Vc, Vc and C1/C2 following capsaicin injection into the whisker pad skin. The number of pERK-IR in the Vi/Vc, Vc and C1/C2 of GluR2 or GluR3 Δ7KI mice and wild type mice 5 (A) and 40 min (B) after capsaicin injection into the whisker pad skin. (TIF) [file pone.0044055.s002.tif]
